# Expression of Tenascin C, EGFR, E-Cadherin, and TTF-1 in Medullary Thyroid Carcinoma and the Correlation with RET Mutation Status

**DOI:** 10.3390/ijms17071093

**Published:** 2016-07-09

**Authors:** Florian Steiner, Cornelia Hauser-Kronberger, Gundula Rendl, Margarida Rodrigues, Christian Pirich

**Affiliations:** 1 Department of Pathology, Paracelsus Medical University Salzburg, Müllner Hauptstrasse 48, A-5020 Salzburg, Austria; f.steiner@salk.at (F.S.); c.hauser-kronberger@salk.at (C.H.-K.); 2 Department of Nuclear Medicine and Endocrinology, Paracelsus Medical University Salzburg, Müllner Hauptstrasse 48, A-5020 Salzburg, Austria; g.rendl@salk.at (G.R.); rodriguesradischat@hotmail.com (M.R.)

**Keywords:** tenascin C, epidermal growth factor receptor (EGFR), E-cadherin, thyroid transcription factor-1 (TTF-1), medullary thyroid carcinoma

## Abstract

Tenascin C expression correlates with tumor grade and indicates worse prognosis in several tumors. Epidermal growth factor receptor (EGFR) plays an important role in driving proliferation in many tumors. Loss of E-cadherin function is associated with tumor invasion and metastasis. Thyroid transcription factor-1 (TTF-1) is involved in rearranged during transfection (RET) transcription in Hirschsprung’s disease. Tenascin C, EGFR, E-cadherin, TTF-1-expression, and their correlations with RET mutation status were investigated in 30 patients with medullary thyroid carcinoma (MTC) (*n* = 26) or C-cell hyperplasia (*n* = 4). Tenascin C was found in all, EGFR in 4/26, E-cadherin in 23/26, and TTF-1 in 25/26 MTC. Tenascin C correlated significantly with tumor proliferation (overall, *r* = 0.61, *p* < 0.005; RET-mutated, *r* = 0.81, *p* < 0.01). E-cadherin showed weak correlation, whereas EGFR and TTF-1 showed no significant correlation with tumor proliferation. EGFR, E-cadherin, and TTF-1 showed weak correlation with proliferation of RET-mutated tumors. Correlation between TTF-1 and tenascin C, E-cadherin, and EGFR was *r* = −0.10, 0.37, and 0.21, respectively. In conclusion, MTC express tenascin C, E-cadherin, and TTF-1. Tenascin C correlates significantly with tumor proliferation, especially in RET-mutated tumors. EGFR is low, and tumors expressing EGFR do not exhibit higher proliferation. TTF-1 does not correlate with RET mutation status and has a weak correlation with tenascin C, E-cadherin, and EGFR expression.

## 1. Introduction

Medullary thyroid carcinoma (MTC) may arise sporadically in about 75% of cases or as part of multiple endocrine neoplasia type 2 (MEN2) syndrome in 20%–25% of cases [1]. MEN 2 syndromes are caused by activating mutations of the proto-oncogene rearranged during transfection (RET) [2]. On the other hand, a loss of function mutation of RET leads to Hirschsprung’s disease [3].

Tenascin C is an extracellular glycoprotein complex expressed by a variety of cells including epithelial, stromal, and tumor cells [4]. It is overexpressed in a wide variety of tumors including gliomas, where it was originally discovered [5]. In most cases, the expression of tenascin C correlates with the tumor grade and is indicative of a worse prognosis [6]. Koperek et al. [7] found tenascin C expression in medullary microcarcinoma and C-cell hyperplasia and suggested that stromal tenascin C expression seems to be an indicator of a further step in carcinogenesis of MTC, irrespective of a RET germ-line mutation.

Mutations of epidermal growth factor receptor (EGFR) have been found in several tumor entities including gliomas, breast cancer, and non-small lung cancer [8]. In the case of MTC, mutations are rarely found, and their significance is unknown [9]. Rodríguez-Antona et al. [9] showed that EGFR overexpression in MTC is seen in as many as 13% of tumors and that metastases show stronger positivity than primary tumors. Furthermore, EGFR overexpression is linked to RET activation. However, in the presence of RET, EGFR does not appear to play an important role in signaling [10].

Loss of function of the molecule E-cadherin in tumors is associated with invasion and metastasis [11,12]. Naito et al. [13] found that expression of E-cadherin was reduced or absent in 50% or more of thyroid cancer cases, and concluded that this loss of E-cadherin expression may be involved in regional lymph node metastasis and in malignant potential of thyroid neoplasms.

Thyroid transcription factor-1 (TTF-1) is involved in gene expression of thyroperoxidase [14] and thyreoglobulin [15]. TTF-1 expression is seen in follicular cell neoplasms [16] as well as in MTC [17]. In the parafollicular cells of MTC, TTF-1 modulates the activity of genes involved in calcium homeostasis [18]. It was recently shown that TTF-1 is also involved in the transcription of human RET in Hirschsprung’s disease [19].

In MTC, the Ki-67 index correlates with the stage of the disease [20]. Primary tumors that had metastasized were found to have higher Ki-67 indices than primary tumors that had not metastasized. Recurrent lymph node metastases were shown to have higher Ki-67 indices than the primary tumors. The Ki-67 index can therefore be used as a prognostic marker in MTC.

In this study, we investigated the expression of tenascin C, EGFR, E-cadherin, and TTF-1 in MTC, and their correlation with RET mutation status. Furthermore, EGFR mutation status in MTC was evaluated.

## 2. Results

**Figure 1:**
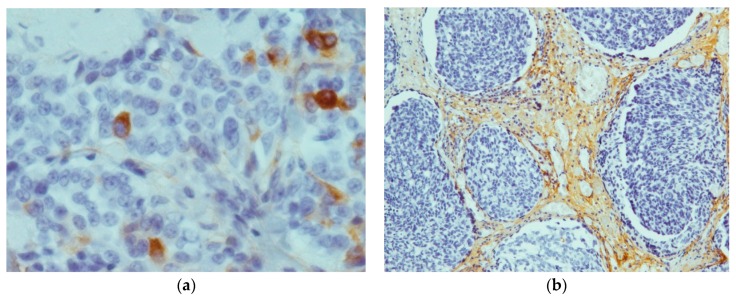
Staining results of tenascin C: (**a**) 40× magnification, showing cytoplasmic staining; (**b**) 10× magnification, depicting staining of the extracellular matrix and the lymph follicle-like accumulation of tumor cells.

Tenascin C showed positive staining results in all the 26 tumors (Figure 1). In contrast, all four cases of C-cell hyperplasia stained negative for tenascin C. The tumor-staining pattern was homogeneously located in all areas of the tumor. However, 14 out of 26 tumors showed expression in the whole tumor field, with the remaining 12 tumors showing partial expression. Except for one case that showed much stronger staining in the periphery, no predominance for tumor center or invasion front could be detected.

Expression of tenascin C was primarily located in the extracellular matrix but also in the plasma membrane and the cytoplasm of parafollicular cells. In areas of lymphocyte infiltration expression levels of tenascin C were particularly high. In non-pathological areas, staining was observed in endothelial cells of blood vessels. The average immunoreactivity score for tenascin C staining was 4.69 ± 2.18. The score ranged from 0 (all negative stained samples) to a maximum score of 7.5.

**Figure 2:**
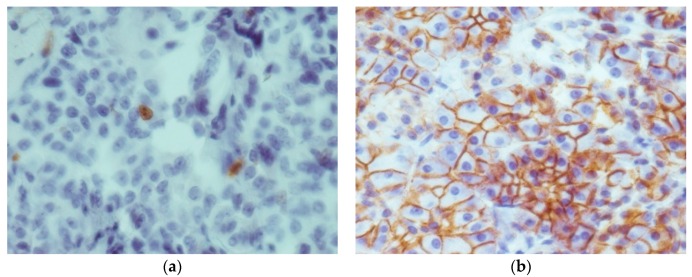
(**a**) 40× magnification, depicting the staining results of Ki-67 (note the proliferating cell in the center); (**b**) 40× magnification, showing strong plasma-membrane expression of E-cadherin.

Staining with E-cadherin showed positive expression in 27 out of 30 cases. However, highest expression was observed in three cases of C-cell hyperplasia, while one case of C-cell hyperplasia showed no staining. Expression of E-cadherin was particularly high in the thyroid follicles. Altogether staining in all areas of the tumor was observed in 15 cases of MTC, with 8 MTC cases showing partial expression and 3 MTC cases showing no expression at all. There were no significant differences in E-cadherin expression between MTC and C-cell hyperplasia. As expected form a membrane bound protein, E-cadherin expression was primarily observed in the plasma membrane of cells (Figure 2). Immunoreactivity scores in E-cadherin samples ranged from 1 to 9 (full range) with a mean score of 4.69 ± 2.4.

**Figure 3:**
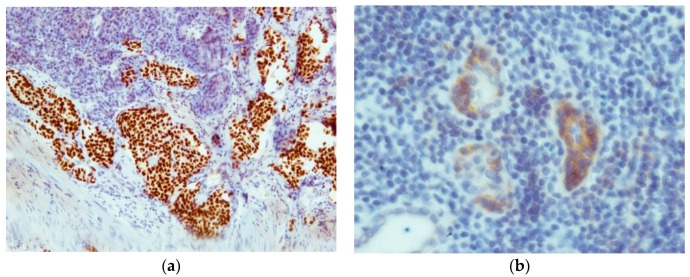
(**a**) 10× magnification, showing thyroid transcription factor-1 (TTF-1) expression in a metastasis in a Meckel’s diverticulum; (**b**) 40× magnification, illustrating epidermal growth factor receptor (EGFR) expression in follicular cells scattered between medullary thyroid carcinoma (MTC) cells.

EGFR expression was very weak, with six positively stained cases consisting of four cases of MTC and two cases of C-cell hyperplasia. Staining was primarily found in the cytoplasm of cells and endothelial cells. The highest staining intensity was found in non-neoplastic follicular cells scattered amid the tumor mass (Figure 3). Inside the tumor area, staining was relatively weak. The mean immunoreactivity score was 1.58 ± 1.20.

With exception of the metastasis to the adrenal gland all tissue samples, including the metastasis in a Meckel’s diverticulum showed TTF-1 expression (Figure 3). The entire tumor area and all of the follicles showed strong staining with the TTF-1 antibody. The samples only differed in the staining intensity, which was moderate to strong. The mean immunoreactivity score was 7.77 ± 1.89.

The staining results for the proliferation marker Ki-67 showed positive staining results in all 30 samples. As expected, protein expression was only seen in the nucleus (Figure 2). Immunoreactivity scores ranged from 1.5 to 6.75 after correction with the correlation coefficient. The mean score was 3.35 ± 1.57. The four cases of C-cell hyperplasia had the lowest Ki-67 expression (*p* < 0.001) with only 1–2 cells per high power field. Tenascin C expression correlated moderately to strongly with the level of the proliferation marker Ki-67 in the tumor tissue. A weak correlation could be observed with E-cadherin, whereas EGFR and TTF-1 showed no significant correlation (Table 1).

All 15 tumors that showed RET mutation were analyzed regarding their expression of tenascin C, EGFR, E-cadherin, and TTF-1. They were then correlated with the proliferation marker Ki-67. Tenascin C expression showed a very strong correlation with the proliferation of RET-mutated tumors, while EGFR, E-cadherin, and TTF-1 showed a very weak correlation (Table 1).

The group of RET-mutated tumors was then split in germ-line-mutated (*n* = 7) and somatic-mutated (*n* = 8) tumors. Expression profiles of both groups were then correlated with proliferation in those tumors. In the case of the germ-line-mutated tumors, tenascin C expression correlated highly (*r* = 0.86) with proliferation. A weak correlation could be observed with E-cadherin and TTF-1 (*r* = −0.26 and −0.33, respectively), whereas EGFR only showed a very weak correlation (*r* = −0.11). In the case of MTC with somatic RET mutation, tenascin C still showed a moderate-to-strong correlation with proliferation (*r* = 0.67). EGFR correlation with proliferation was moderate (*r* = 0.51), while E-cadherin and TTF-1 showed low (*r* = 0.39) and very low correlations (*r* = 0.02), respectively.

MTC with RET wild-type were also investigated. Tenascin C showed a very weak correlation, E-cadherin a weak to moderate correlation, and TTF-1 a strong correlation with tumor proliferation (Table 1). EGFR analysis was not performed in this group because none of the specimens showed positivity for EGFR. Only EGFR expression differed significantly between RET-mutated and RET wild-type tumors (*r* = 0.51, *p* = 0.001). Tenascin C, E-cadherin, and TTF-1 did not differ in their respective expression levels.

RET-mutated and wild-type tumors were compared to evaluate whether the mutation status of RET affects TTF-1 expression. No significant difference in TTF-1 expression was found between both groups. RET-mutated MTC showed no correlation with TTF-1 expression for germ-line- and somatic-mutated tumors (*r* = −0.33 and 0.02, respectively, *p*-value is not significant (*p* ns)). TTF-1 expression correlated with tenascin C, EGFR, and E-cadherin expression. Tenascin C correlation was very weak (*r* = −0.10, *p* ns), while the EGFR and E-cadherin correlation was weak (*r* = 0.37 and 0.21, respectively, *p* ns).

EGFR positively stained tumors (all RET wild-type) did not show a significantly higher Ki-67 index, as compared with EGFR negatively stained tumors.

A weak to moderate correlation (*r* = 0.08–0.40, *p* ns) between calcitonin levels and Ki-67 was found. Preoperative calcitonin levels only showed a weak correlation with tenascin C expression (*r* = 0.18, *p* < 0.05) and Ki-67 (*r* = 0.10, *p* ns). Post-operative calcitonin levels correlated moderately with tenascin C expression (*r* = 0.53, *p* < 0.005) and Ki-67 (*r* = 0.40, *p* ns). Except for the inverse correlation for EGFR (*r* = −0.38, *p* < 0.05), post-operative calcitonin levels showed weak to no correlation with E-cadherin, TTF-1, and EGFR expression (*r* = 0.22, 0.08 and 0.07, respectively, *p* ns).

Both pre-operative and post-operative calcitonin levels were not significantly different between RET-mutated and wild-type tumors.

## 3. Discussion

In this study, we found tenascin C expression in all MTC, but in none of the C-cell hyperplasia cases. Tenascin C was primarily located in the stromal areas of tumors, but could also be detected in the cytoplasm and plasma membrane. Our results are in agreement with the findings by Koperek et al. [7] of tenascin C expression in all cases of MTC and in only 52% of C-cell hyperplasia cases. The difference in tenascin C expression in C-cell hyperplasia is most likely due to the larger study group used by Koperek et al. Our study cohort included only 30 patients because of the rarity of the disease. Furthermore, we investigated only four C-cell hyperplasia and two MTC metastases. The relationship between tenascin C expression and tumor proliferation needs to be further investigated. It seems that RET mutation is associated with a higher level of tenascin C expression, even though we found no significant difference between RET-mutated and wild-type MTC. It might be that, with a larger study cohort, a significant difference between RET-mutated and wild-type MTC could be established. Furthermore, it seems that the bc-24 clone used for tenascin C staining does not uniquely bind to tenascin C, but to other tenascin subtypes over the EGF-like repeats. Therefore, the results might not solely represent tenascin C, but also the expression of other tenascin isoforms.

EGFR plays an important role in driving proliferation in a variety of tumors [8]. In the study of Rodriguez-Antona et al. [9], EGFR expression was shown in a subset of 18 tumors, and it was thus concluded that EGFR might be a target for drug therapy. We therefore evaluated if MTC expresses EGFR and, if so, to what degree. In our study, EGFR expression could be detected in six cases (15%), with few staining cells and scattered expression. Our results are consistent with the reported EGFR expression of 9% and 35% in primary MTC and metastasis, respectively. Additionally, it seems that EGFR expression is significantly higher in MTC carrying a RET mutation [9]. Due to these reports, we performed EGFR mutation analysis on three cases with the highest EGFR expression. However, no mutations could be detected using the Cobas^®^ EGFR mutation analysis kit. Our results are consistent with the finding by Rodriguez-Antona et al. [9] of nucleotide changes of unknown significance in only one sample. It thus seems that, although some EGFR expression can be detected, the role of EGFR in MTC is of a minor nature. Therefore, the absence of activating mutations questions the use of EGFR inhibitor drugs. This suggestion is further backed by Vitagliano et al. [21], who found that EGFR downstream signaling is of minor significance in the presence of active RET.

We also looked at the expression of E-cadherin, a plasma membrane protein important for cell–cell adhesion [11,12]. In our findings, E-cadherin showed staining in 26 cases of MTC (87%). The remaining samples showed no staining including one case of C-cell hyperplasia. Naito et al. [13] reported that low E-cadherin expression was associated with a higher malignant potential as well as regional lymph node metastasis. We therefore compared the expression levels of E-cadherin in C-cell hyperplasia and MTC, which showed no statistical significance. This is probably due to the small number of cases with C-cell hyperplasia in our study cohort.

In our study, with the exception of a metastasis in the adrenal gland, TTF-1 staining was moderate to strong in all tissue samples. We also found TTF-1 expression in a metastasis in a Meckel’s diverticulum. These data seem to indicate that TTF-1 can be used as a useful marker for detecting primary MTC or metastasis, as previously suggested by Katho et al. [17].

The expression of the proliferation marker Ki-67 was generally low in our study cohort. As expected, C-cell hyperplasia showed the lowest Ki-67 indices, which were significantly lower than those found in MTC. Ishihara et al. [22] reported that breast cancers staining positive for tenascin carried a less favorable prognosis. We therefore evaluated if the expression of tenascin C in MTC correlates with tumor proliferation. We found that the Ki-67 index correlated moderately to strong with tenascin C expression. It might therefore be that tenascin C expression can be used as a marker for the malignant potential of a MTC. On the other hand, we observed that E-cadherin shows weak inverse correlation to tumor proliferation. As previously found by Naito et al. [13], low E-cadherin expression correlates with higher malignant potential of the tumor. This might also be true for our study group, but the size of our cohort may be a limiting factor.

The RET proto-oncogene is an important molecule in the development of MTC. We investigated if RET mutation correlates with a higher expression of tenascin C, EGFR, E-cadherin, or TTF-1. Furthermore, we evaluated whether proliferation is higher in RET-mutated MTC. We found that tenascin C expression in RET-mutated tumors showed a high correlation to proliferation. However, except for a significantly higher degree of EGFR expression in RET wild-type tumors, no significant difference in the expression of E-cadherin or TTF-1 could be detected between RET-mutated and wild-type MTC. Rodriguez-Antona et al. [9] also found that EGFR expression was higher in RET-mutated tumors, depending on the localization of the mutation.

We thereafter investigated RET-mutated tumors where the mutation was germ-line-derived or a somatic mutation. The expression profiles of the tumors in each group were then correlated with the proliferation marker Ki-67. Tenascin C correlated highly to proliferation in the germ-line-mutated group, whereas EGFR, E-cadherin, and TTF-1 showed a weak correlation. In the somatic-mutated tumors, tenascin C correlation was lower but showed a higher correlation to EGFR.

Calcitonin has proven to be a useful marker in the diagnosis and prognosis of MTC [23]. We found that both basal and pentagastrin stimulated calcitonin levels did not differ significantly between RET-mutated and wild-type MTC. Furthermore, no correlation between basal calcitonin levels and the Ki-67 index, tenascin C, EGFR, E-cadherin, or TTF-1 was observed. A moderate correlation was found between post-operative calcitonin levels and both Ki-67 index and tenascin C expression. However, due to the low level of correlation, it is possible that these results are stochastic.

The role of TTF-1 in the development of Hirschsprung’s disease by RET interaction has been recently outlined [19]. Furthermore, not only papillary thyroid carcinoma but also MTC show expression of TTF-1 [17]. Garcia-Barcelò et al. [24] found that mutations in single nucleotide polymorphisms (SNPs) of NKX2 (codes for TTF-1) and the RET promoter region correlated with the decreased TTF-1 binding and activation of RET, leading to Hirschsprung’s disease. It is known that a loss of RET activation leads to Hirschsprung’s disease [3], whereas a gain in function leads to MTC [2]. It is possible therefore that TTF-1 expression in RET-mutated MTCs might be higher, leading to consecutive RET activation. However, we found no significant difference in TTF-1 levels between RET-mutated (germ-line- and somatic-mutated) and RET wild-type tumors. Our data seem thus to indicate that TTF-1 does not play a role in the consecutive activation of RET. Moreover, we observed that TTF-1 has a weak correlation with EGFR and E-cadherin, but no correlation with tenascin-C or the Ki-67 index. However, the role of TTF-1 in MTC has yet to be established by a study with a larger cohort.

## 4. Materials and Methods

In the present study, 30 patients (16 females, 14 males; age: 2–81 years, mean age: 51 ± 18 years) with diagnosed MTC (*n* = 26) or C-cell hyperplasia (*n* = 4) at the Medical University of Salzburg were investigated. Eight patients showed MEN (MEN2A, 7 patients; MEN2B, 1 patient). All subjects gave their informed consent for inclusion before they participated in the study. The study was conducted in accordance with the Declaration of Helsinki, and the protocol (Approval: 14 February 2014) was approved by an institutional review board.

Routinely performed formalin-fixed paraffin embedded (FFPE) tissue was obtained from the primary thyroid site in 22 patients, lymph node metastasis in 6 patients, metastasis in a Meckel’s diverticulum in 1 patient, and metastasis in the adrenal gland in 1 patient.

Genetic analysis of RET mutations was carried out in 21 patients, 6 of them with MEN2. RET gene mutations were detected in 15 patients (Table 2), while 6 patients showed RET wild-type.

Preoperative serum calcitonin levels (2.2–3293.4 ng/L, mean: 596.4 ng/L) were measured in 23 patients and pentagastrin tests (calcitonin: 17.7–2936.7 ng/L; mean: 708.3 ng/L) were performed in 11 patients. At time of the study, serum calcitonin levels (0.7–289,951.0 ng/L, mean: 11,056.6 ng/L) and pentagastrin test results (calcitonin: 2.6–971.7 ng/L; mean: 188.9 ng/L) were available in 29 patients and 16 patients, respectively. The normal calcitonin levels were <15 ng/L for males and <5 ng/L for females.

The expression of tenascin C, EGFR, E-cadherin, TTF-1, and Ki-67 was evaluated by immunohistochemistry. The primary antibodies used, with the working dilutions and pH of antigen retrieval buffers, are listed in Table 3.

EGFR mutation analysis was performed using the Roche™ Cobas^®^ EGFR mutation kit (Roche Molecular Systems, Inc., Branchburg, NJ, USA) on a Cobas^®^ 4800 platform, v2.0 (Roche Molecular Systems, Inc.).

### Statistical Analysis

Excel^®^ software (Microsoft Corporation, Vienna, Austria) was used for the statistical evaluation of results.

Correlation analysis of tenascin C, EGFR, E-cadherin, and TTF-1 with the Ki-67 index was done by using the Pearson correlation coefficient test. For the assessment of statistical significance, the *t*-test for unpaired variance was used. Statistical significance was defined as *p* < 0.05.

## 5. Conclusions

MTC express tenascin C, E-cadherin, and TTF-1. Tenascin C expression correlates significantly with tumor proliferation, especially in RET-mutated tumors. EGFR expression is low in MTC and tumors showing EGFR expression do not exhibit higher proliferation. However, EGFR expression is significantly higher in MTC with RET mutation. No EGFR mutation was found in MTC. TTF-1 expression does not correlate with RET mutation status. TTF-1 expression has a weak correlation with tenascin C, E-cadherin, and EGFR expression.

## Figures and Tables

**Table 1:** Correlation of tenascin C, EGFR, E-cadherin, and TTF-1 expression with the proliferation marker Ki-67.

MTC	Tenascin C	EGFR	E-Cadherin	TTF-1
Overall MTC				
*r*-value	0.61	−0.04	−0.19	0.13
*p*-value	<0.005	ns	<0.05	ns
RET-mutated MTC				
*r*-value	0.81	0.14	−0.11	−0.12
*p*-value	<0.01	ns	ns	ns
Wild-type MTC				
*r*-value	0.08	–	−0.40	0.72
*p*-value	ns	–	ns	<0.001

EGFR: epidermal growth factor receptor; TTF-1: thyroid transcription factor-1; MTC: medullary thyroid carcinoma; RET: rearranged during transfection; *r*: Pearson correlation coefficient; *p*: probability of obtaining a positive test result; ns: not significant.

**Table 2:** Rearranged during transfection (RET) mutations detected in the study group.

Mutation Detected	Sporadic MTC/MEN2
Codon 769 on Exon 13 (*n* = 5)	Sporadic
Codon 904 on Exon 15 (*n* = 3)	Sporadic
Codon L790F on Exon 13 + Codon 769 on Exon 13 (*n* = 3)	MEN2A (familial)
Codon L790F on Exon 13 + Codon 904 on Exon 15 (*n* = 1)	MEN2A
Codon 790 on Exon 13 (*n* = 1)	MEN2A
Codon 634 on Exon 11 (*n* = 1)	MEN2A
Codon 836 on Exon 14 (*n* = 1)	Sporadic

*n*, number of patients; MEN, multiple endocrine neoplasia.

**Table 3:** List of primary antibodies, working dilutions and pH of antigen retrieval buffers used.

Antibody	Source	Clone	Type	Species	pH-Retrieval	Working Dilution
Tenascin C	Sigma Aldrich™	bc-24	mc	Mouse	pH 6	1:4000
	Santa Cruz™	bc-24	mc	Mouse	pH 6	1:4000
EGFR	Dako™	E30	mc	Mouse	pH 6	1:20
E-Cadherin	Thermo Scientific™	SPM471	mc	Mouse	pH 9	1:100
TTF-1	Novocastra™	SPT24	mc	Mouse	pH 9	1:50
Ki-67	Dako™	MIB-1	mc	Mouse	pH 9	1:500

EGFR: epidermal growth factor receptor; TTF-1: thyroid transcription factor-1; mc: monoclonal antibody.
